# Real-time monitoring of intravenous thrombolysis in acute ischemic stroke using rotational thromboelastometry: a feasibility pilot study

**DOI:** 10.1007/s00415-022-11271-z

**Published:** 2022-07-19

**Authors:** Alexander Tinchon, Elisabeth Freydl, Robert D. Fitzgerald, Christina Duarte, Michael Weber, Bernadette Calabek-Wohinz, Christoph Waiß, Stefan Oberndorfer

**Affiliations:** 1 Karl Landsteiner University of Health Sciences, Dr. Karl-Dorrek-Straße 30, 3500 Krems, Austria; 2 Department of Neurology, University Hospital St. Pölten, Dunant-Platz 1, 3100 St. Pölten, Austria; 3 Karl Landsteiner Institute of Clinical Neurology and Neuropsychology, University Hospital St. Pölten, Dunant-Platz 1, 3100 St. Pölten, Austria; 4 Karl Landsteiner Institute of Anesthesiology and Intensive Care Medicine, Clinic of Hietzing, Wolkersbergenstrasse 1, 1130 Vienna, Austria; 5 Department of General Health Studies, Division Biostatistics and Data Science, Karl Landsteiner University of Health Sciences, Dr. Karl-Dorrek-Straße 30, 3500 Krems, Austria

**Keywords:** Viscoelastometry, ROTEM, Stroke, Intravenous thrombolysis, Coagulation, Point-of-care assessment

## Abstract

**Introduction:**

Rotational thromboelastometry (ROTEM) records whole blood coagulation in vitro. Data on dynamic changes of clot patterns during intravenous thrombolysis (IVT) in acute ischemic stroke is scarce. We investigated the feasibility of ROTEM as a potential point-of-care assessment tool for IVT.

**Methods:**

In this prospective pilot study, patients with acute stroke symptoms received IVT. Whole blood coagulation was tracked on the ROTEM analyzer. Blood samples were analyzed before, and then 2, 15, 30 and 60 min after beginning IVT. In vitro clots (iCLs) were described by their maximum clot firmness (MCF), the time needed to reach MCF (MCF-t), as well as the area under the curve (AR10). National Institutes of Health Stroke Scale (NIHSS) was used as early clinical outcome parameter.

**Results:**

We analyzed 288 iCLs from 12 patients undergoing IVT. In all iCLs, an early fibrinolysis (91% within the first 10 min) was detected during IVT. Three different curve progression patterns were observed: a low-responder pattern with a continuous clot increase, a high-responder pattern with a sustained clot decrease or total clotting suppression and an intermediate-responder pattern with alternating clot characteristics. There was a difference among these groups in early clinical outcome (AR10 and MCF each *p* = 0.01, MCF-t *p* = 0.02, Kruskal–Wallis Test).

**Conclusion:**

The fibrinolysis patterns determined using ROTEM allow for the monitoring of IVT in patients with acute ischemic stroke. This pilot study found a correlation between the in vitro fibrinolysis patterns and early clinical outcomes. These findings support a potential for individualization of IVT in the future.

**Supplementary Information:**

The online version contains supplementary material available at 10.1007/s00415-022-11271-z.

## Introduction

Mechanical thrombectomy and IVT are considered the gold standard therapies for acute ischemic stroke [1–3]. Clinical response to IVT varies. Some patients recover while others show no benefit [4, 5]. Therefore, an individualization of the fibrinolytic therapy based on the patient’s response to IVT may prove useful. Until now, no suitable tool has been established in clinical routine, which could help distinguish between high and low responders to IVT.

Viscoelastometry (VEM) measures whole blood coagulation in vitro. Initially reported in 1948, VEM was developed to provide a quick overview of the coagulation status as a whole in vitro [6]. It has the advantage of being able to serve as a bedside monitoring test in terms of a point-of-care assessment [7]. Viscoelastic assays therefore are already in successful clinical use for the evaluation of blood transfusion requirements during cardiac surgery, trauma-induced coagulopathy and coagulation monitoring in liver transplantation [8–11]. The most frequently used VEM test systems are thromboelastography (TEG) and rotational thromboelastometry (ROTEM) [12]. ROTEM measures changes in blood viscosity by movement of a pin through whole blood in a fixed cup. The rotation of the pin is inversely related to the clot strength, which subsequently is translated into a typical clot graph by a charge-coupled device image sensor system [13, 14].

Although ROTEM’s clinical main area of application is point-of-care assessment in anesthesiology, it has also been used to evaluate whole blood coagulation in patients with ischemic stroke [15–20]. However, both main objectives and study designs differed and no dynamic coagulation profiling with outcome prediction has yet been performed.

IVT induces hyperfibrinolysis, which can be detected using ROTEM [17, 19, 20].

We addressed two main questions: can changes be detected in ROTEM during IVT, which may be predictive of clinical outcome? If so, what are the ideal test channels and time points of measurement? To find out, we performed a hypothesis-generating feasibility study.

## Methods

Patients with acute stroke symptoms who underwent IVT were included in this prospective exploratory pilot study. Treatment with mechanical thrombectomy was an exclusion criterion in order to ensure comparability in the outcome analysis. Patients with early signs of infarction in the CT or FLAIR positivity in the MRI were excluded. Patients were included consecutively, whenever testing capacities and personnel was available. Written informed consent was obtained from patients or, in case of mental disability, from their legal representatives. The study was approved by the local institutional ethics board (Ref: GS1-EK-4/338/2015). All patients received alteplase according to the recent guidelines of the American Heart Association/Stroke Association [21]. Ischemic stroke was confirmed by MRI or CT scan prior to or after IVT.

Whole blood coagulation and fibrinolysis were recorded on the ROTEM^®^ Whole Blood Hemostasis System Type Delta as instructed by the manufacturer. In order to identify the most suitable test channel for outcome correlation, every measurement was carried out as 4-channel analysis, comprising: extrinsic activation of coagulation (EXTEM), intrinsic activation of coagulation (INTEM), fibrin fraction of the clot after platelet inhibition with cytochalasin D (FIBTEM) and a control test after plasmin inhibition with aprotinin (APTEM); as this channel reverses the plasmin effect in vitro, it was not used for outcome correlation.

Blood samples were taken by venous puncture in a citrate tubule (VACUETTE^®^ 9NC Coagulation Sodium Citrate 3.2%). 4-channel analysis was performed before (baseline), and then at 4 testing time points during IVT: post bolus (2 min), 15 min, 30 min, and 60 min. All samples were processed immediately after, or at least within 2 h after venous puncture. An additional follow-up test was performed 18 to 36 h after IVT. The ROTEM device was located at the stroke unit, allowing for rapid testing in terms of a point-of-care assessment. The latency between blood sampling and the start of in vitro testing was limited to a couple of minutes. In order to reliably record the whole coagulation process (clot formation and lysis) in vitro, every measurement (4-channel analysis) at any testing time point was scheduled for at least 20 min (= ”running time”).

The following ROTEM parameters were recorded to describe the iCLs at each measurement:

- CT (clotting time),
- CFT (clot formation time),
- MCF (maximum clot firmness),
- MCF-t (time until formation of MCF) and
- AR5-AR20 (area under the curve from CT to 5–20 min after beginning of in vitro testing).

**Fig. 1:**
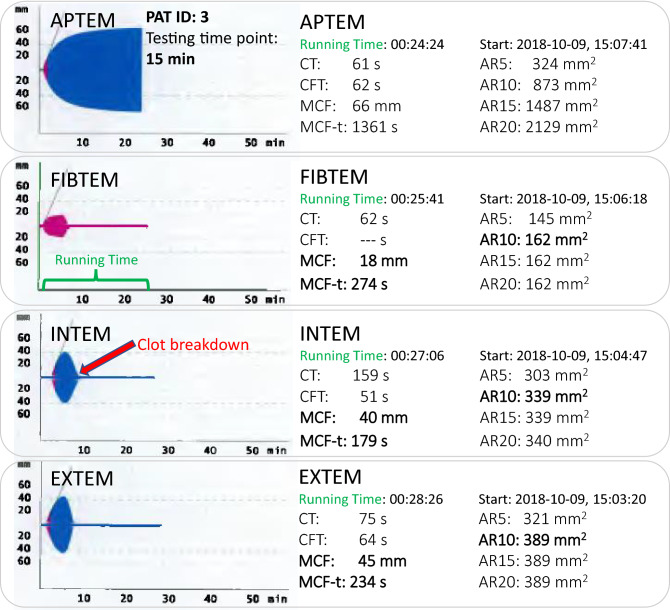
Example of a 4-channel analysis at a 15-min testing time point after starting IVT, patient ID: 3. The test channels APTEM, FIBTEM, INTEM and EXTEM are displayed top down. In vitro clots are characterized by the ROTEM parameters CT, CFT, MCF, MCF-t, AR5-AR20, shown on the right side. Y-axis: clot amplitude in mm; X-axis: running time in minutes (green letters). MCF, MCF-t and AR10 (main target parameters) are written in bold. Note that AR15 and AR20 do not differ from AR10, because clot breakdown (red arrow) occurred within 10 min of running time

After starting the analysis by activating the extrinsic or intrinsic coagulation system in vitro, CT elapses at zero line until clot formation begins to be visible as a typical clot graph. The duration from this time point until reaching an iCL amplitude of 20 mm is expressed as CFT. The iCL amplitude further increases until reaching its maximum (MCF). Subsequently iCL firmness rapidly decreases due to fibrinolytic therapy and then returns to the zero line (= clot breakdown). See Fig. 1 for an example of a 4-channel analysis at the 15-min testing time point after beginning IVT and Figure S1 (Supplemental Material) for a standard ROTEM curve without therapeutical intervention.

**Fig. 2:**
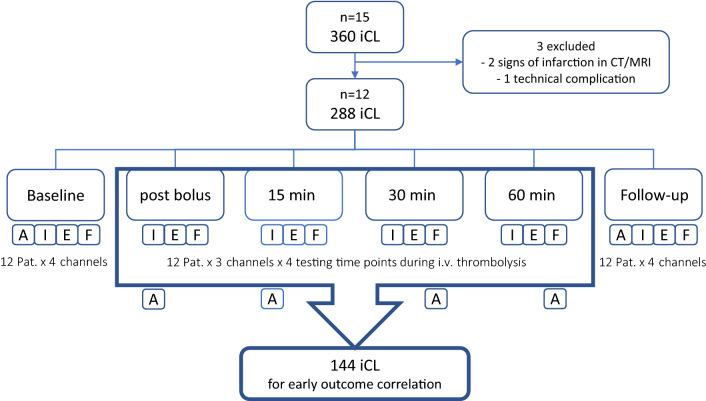
Flow diagram of the recruiting process. After excluding 3 patients, 288 in vitro clots remained for final analysis. 4-channel-analysis: *A* APTEM, *I* INTEM, *E* EXTEM, *F* FIBTEM. Baseline and follow-up measurements, as well as the test channel APTEM were excluded from correlation with early clinical outcome, because they served as controls and do not reflect the fibrinolytic response to IVT

iCLs of the test channels INTEM, EXTEM and FIBTEM during IVT (four testing time points: post bolus, 15, 30, and 60 min) were used for the correlation with early clinical outcome. APTEM, as well as baseline and follow-up iCLs were not included in the analysis, because they served as controls and did not reflect a response to IVT. See Fig. 2 for a flow diagram of the recruiting process.

Increase or decrease in ROTEM parameters between two consecutive testing time points during IVT resulted in different curve progression patterns. Absolute measuring values at each testing time point as well as a comparison of curve progression patterns were used for the correlation with early clinical outcome.

National Institutes of Health Stroke Scale (NIHSS) was used as clinical outcome parameter and recorded before and at follow-up by an independent investigator [22]. A favorable clinical outcome was considered an NIHSS improvement of at least four points. Unfavorable clinical outcome was defined as no improvement or reduction of max. 1 point in NIHSS.

### Statistics

Descriptive statistics of ROTEM parameters were drawn up and results were correlated to the clinical outcome. Non-parametric Kruskal–Wallis Test was used to compare clinical outcome between different groups of fibrinolytic response. Spearman’s rank correlation coefficient *ρ* was used to analyze data conformity between the different ROTEM test channels (INTEM, EXTEM and FIBTEM).

## Results

We obtained 360 single iCLs from 15 alteplase-treated patients from August 2018 to March 2019. Three patients were excluded from final analysis: two of them had signs of infarction in the initial cerebral imaging (corticomedullary dedifferentiation and FLAIR positivity) and one of them had missing data due to technical complications, leaving 288 iCLs for final analysis (12 patients × 4 channels × 6 testing time points = 288). One hundred forty-four iCLs (*n* = 144) were used for the correlation with early clinical outcome (12 patients × 3 channels × 4 testing time points = 144; see Fig. 2).

Median age was 76 years, gender ratio was 1.5:1 (m/f). All iCLs (*n* = 144, 100%) showed a complete breakdown within test running times of at least 20 min at every single testing time point during IVT (55% within 5 min, *n* = 79; 36% within 10 min, *n* = 52; 6% within 15 min, *n* = 8; 3% within 20 min, *n* = 5).

Compared to IVT, all parameters fully recovered in the follow-up test. Main changes were observed for the parameters maximum clot firmness (MCF), time to maximum clot firmness (MCF-t), and area under the curve after 10 min running time in vitro (AR10), while clotting time (CT) and clot formation time (CFT) did not reveal any relevant changes during IVT. See Table 1 for descriptive statistics of all patients in the test channel INTEM and Table 2 for corresponding clinical stroke characteristics, complications in follow-up imaging and vascular risk factors [23].

**Table 1:** Descriptive statistics for all patients, sorted by responder patterns, test channel: INTEM

	All (*n* = 12)	RED pattern Low responders (*n* = 4)	GREEN pattern High responders (*n* = 4)	BLUE pattern Intermed. resp. (*n* = 4)
Median age (SD)	76.5 (10)	76.5 (9.7)	72.0 (12.2)	76.0 (8.9)
Males, *n* (%)	7 (58)	3 (75)	3 (75)	1 (25)
Median **NIHSS**^a^ BL^b^(range)	4 (1–14)	4.5 (1–14)	6 (4–10)	3 (2–4)
Median **NIHSS** FU^c^ (range)	2 (0–14)	4 (1–14)	2 (0–2)	1.5 (1–2)
Median **MCF** BL, mm (range)	62 (49–76)	61 (54–76)	62 (49–70)	62 (59–71)
Median **MCF** IVT^d^, mm (range)	21 (0–51)	20 (0–51)	12 (0–46)	22 (0–38)
Median **MCF**^e^ FU, mm (range)	63 (57–70)	62 (58–66)	63 (57–70)	64 (58–69)
Median **MCF-t**^f^ BL, sec (range)	1287 (1072–1503)	1206 (1072–1503)	1255 (1200–1299)	1411 (1159–1441)
Median **MCF-t** IVT, sec (range)	119 (0–510)	123 (0–510)	56 (0–261)	141 (0–309)
Median **MCF-t** FU, sec (range)	1340 (1185–2396)	1660 (1361–2052)	1269 (1186–2396)	1229 (1185–1765)
Median **AR-10**^g^ BL, mm^2^ (range)	808 (555–1003)	793 (714–829)	829 (555–927)	825 (717–1003)
Median **AR-10** IVT, mm^2^ (range)	124 (0–810)	125 (0–810)	55 (0–634)	137 (0–395)
Median **AR-10** FU, mm^2^ (range)	856 (715–946)	817 (715–897)	837 (732–946)	870 (760–909)
Median **CT**^h^ BL, sec (range)	154 (121–265)	140 (122–265)	178 (121–189)	154 (140–210)
Median **CT** IVT, sec (range)	152 (47–306)	150 (124–306)	155 (47–175)	163 (110–212)
Median **CT** FU, sec (range)	160 (124–208)	153 (124–160)	183 (151–203)	173 (156–208)
Median **CFT**^i^ BL, sec (range)	77 (43–144)	85 (75–90)	68 (58–144)	71 (43–101)
Median **CFT** IVT, sec (range)	74 (51–254)	73 (58–82)	66 (51–142)	85 (53–231)
Median **CFT** FU, sec (range)	71 (53–90)	68 (56–90)	67 (53–86)	74 (61–83)

^a^National Institutes of Health Stroke Scale; ^b^Baseline; ^c^Follow-up; ^d^Intravenous thrombolysis; ^e^Maximum clot firmness; ^f^Time to maximum clot firmness; ^g^Area under the curve after 10 min in vitro; ^h^Clotting time; ^i^Clot formation time. Reference values: CT 100–240 s, CFT 30–110 s, MCF 50–72 mm, MCF-t depending on test running time, AR-10 depending on CT, CFT and MCF

**Table 2:** Clinical/radiological characteristics of all patients, sorted by responder patterns

Patient ID	Group *RED* (*n* = 4)				Group *GREEN* (*n* = 4)				Group *BLUE* (*n* = 4)			
	1	2	8	13	3	4	10	12	5	7	11	15
Gender	m	m	m	f	m	f	m	m	f	f	m	f
Age	72	77	76	94	85	67	77	57	81	71	64	83
TOAST classification	LAA	CE	SVO	LAA	SVO	SVO	SUE	SOD^c^	SVO	CE	SUE	SUE
*Infarct characteristics*												
Initial cerebral imaging	CT	CT	CT	CT	MRI	CT	CT	CT	MRI	CT	CT	CT
Vascular supply area	MCA	MCA	MCA	MCA	MCA	PCA	MCA	PCA	MCA	ACA	PCA	MCA
Infarct size^a^	< 1/3	< 2/3	< 1/3	< 2/3	< 1/3	< 1/3	no	< 1/3	< 1/3	< 2/3	< 2/3	no
Intracerebral artery occlusion	No	M2	No	No	No	No	No	No	No	No	No	No
Recanalization	n.a	Yes	n.a	n.a	n.a	n.a	n.a	n.a	n.a	n.a	n.a	n.a
*Complications in follow-up imaging*												
Hemorrhagic transformation	No	No	No	No	No	No	No	No	No	No	No	No
Edema	No	No	No	No	No	No	No	No	No	No	No	No
*Vascular risk factors*												
Carotid stenosis	90%	No	60%^b^	90%	No	No	50%	No	No	No	60%	No
Atrial fibrillation	No	Yes	No	no	No	No	No	No	No	Yes	No	No
Arterial hypertension	Yes	Yes	Yes	Yes	Yes	Yes	No	Yes	Yes	Yes	Yes	Yes
Hyperlipidemia	Yes	Yes	Yes	Yes	Yes	Yes	Yes	Yes	Yes	Yes	Yes	Yes
Diabetes mellitus	No	No	Yes	No	Yes	No	No	No	Yes	No	Yes	No
Coronary heart disease	No	No	No	No	Yes	No	No	No	No	No	Yes	Yes
Active smoking	No	No	No	No	No	No	Yes	No	No	No	No	No
Alcohol abuse	No	No	No	No	No	No	Yes	Yes	No	No	No	No

*m* male, *f* female, *LAA* large-artery atherosclerosis, *CE* cardioembolism, *SVO* small-vessel occlusion, *SUE* stroke of undetermined etiology, *SOD* stroke of other determined etiology, *MCA* middle cerebral artery, *PCA* posterior cerebral artery, *ACA* anterior cerebral artery, *n.a.* not applicable/no intracerebral artery occlusion; ^a^extend of infarction in the given vascular supply area; ^b^contralateral to cerebral infarct location, ^c^presumed basilar artery stenosis

The test channels INTEM, EXTEM and FIBTEM showed a strong correlation for all iCL parameters. INTEM and EXTEM exhibited nearly identical changes of ROTEM parameters throughout IVT. FIBTEM showed lower, but still high correlations with INTEM and EXTEM. See Table 3 for correlation of differences between the testing time points 15–30 min and 30–60 min.

**Table 3:** Spearman’s rank correlation coefficient *ρ* for the test channels INTEM, EXTEM and FIBTEM, sorted by the ROTEM parameters AR10, MCF and MCF-t

	Testing time point 15–30 min			Testing time point 30–60 min		
	AR10	MCF	MCF-t	AR10	MCF	MCF-t
INTEM-EXTEM						
*Corr. Coeff. ρ*	0.909	0.982	0.952	0.994	0.981	0.934
INTEM-FIBTEM						
*Corr. Coeff. ρ*	0.791	0.875	0.536	0.639	0.681	0.545
EXTEM-FIBTEM						
*Corr. Coeff. ρ*	0.902	0.925	0.802	0.724	0.591	0.738

Correlations were calculated for differences in total values between the testing time points at 15–30 and 30–60 min after starting IVT

**Fig. 3:**
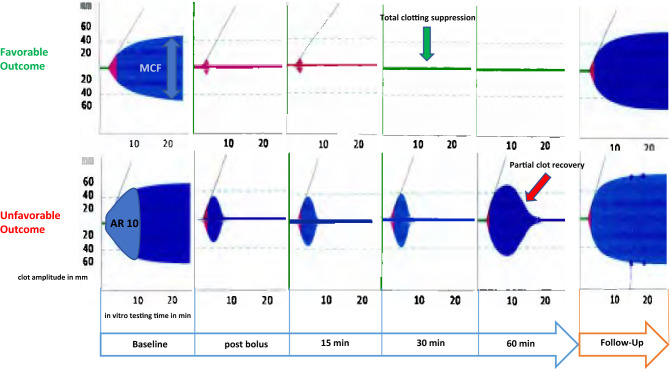
Flowchart, providing examples of a high responder and a low responder in INTEM. A high response to IVT (upper curve progression pattern), indicated by sustained rapid fibrinolysis and/or total clotting suppression (green arrow) was associated with a favorable outcome. A low response to IVT, indicated by gradual reduction of fibrinolysis and partial clot regeneration (lower curve pattern, red arrow) was associated with unfavorable outcome. *MCF* maximum clot firmness, *AR10* area under the curve after 10 min running time. Y-axis: clot amplitude in mm; arrowed X-axis: testing time points during IVT; X-axis below each single measurement: running time for each single measurement

When analyzing the differences between two consecutive testing time points during IVT, three curve progression patterns were identified for the ROTEM parameters MCF, MCF-t and AR10. The *RED* pattern showed a continuous clot increase from 15 to 30 min and 30 to 60 min (= low responders). The *GREEN* pattern exhibited a sustained clot decrease or even a total clotting suppression from 15 to 30 min and 30 to 60 min (= high responders). See Fig. 3 for examples of curve progression patterns in patients with either favorable or unfavorable outcomes. The *BLUE* pattern showed alternating (increasing and decreasing) curve progression patterns. See Table S1 (Supplemental Material) for percentage changes of the parameters MCF, MCF-t and AR10 during IVT. No correlation was found for absolute ROTEM parameters or certain cutoff values at any testing time point during IVT.

**Fig. 4:**
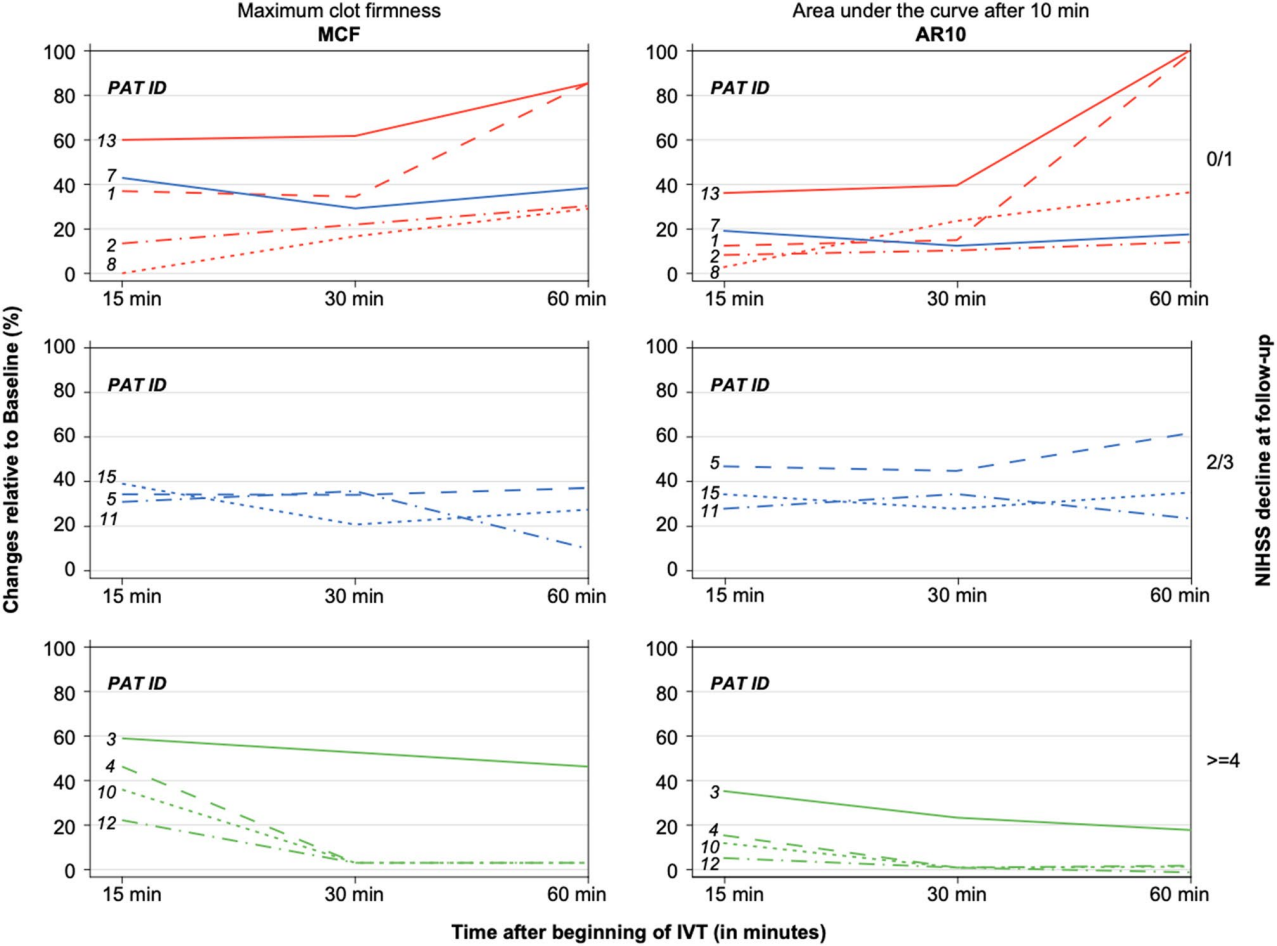
Individual curve progression patterns of all patients (test channel: INTEM), expressed as changes of MCF and AR10 relative to baseline in % (left Y-axis) during IVT (X-axis, testing time points 15, 30 and 60 min after beginning IVT) and sorted by clinical outcome = NIHSS decline at follow-up (right Y-axis, 0/1 unfavorable; 2/3 intermediate;  ≥  4 favorable). The post bolus measurement is not depicted on the X-axis because it uniformly decreased in all patients and therefore did not contribute to the curve progression pattern analysis. Colored lines represent the 3 different ROTEM patterns: *RED*: continuous clot increase (low responders, unfavorable outcome, upper boxes); *GREEN*: continuous clot decrease/total clotting suppression (high responders, favorable outcome, lower boxes); *BLUE*: increasing and decreasing clot patterns (intermediate responders, middle boxes). Note that Patient ID 7 was allocated to the upper boxes, because he/she had a *BLUE* pattern and an unfavorable outcome

There was a difference in clinical outcome among the three patterns (AR10 and MCF each *p* = 0.01, MCF-t *p* = 0.02, Kruskal–Wallis Test). See Fig. 4 for the individual curve progression patterns of all patients at the testing time points 15, 30, and 60 min, sorted by pattern and clinical outcome.

## Discussion

We registered systematic differences in the fibrinolytic response over time during IVT. In the *GREEN* pattern (high responders), fibrinolysis was sustained, while in the *RED* pattern (low responders) fibrinolysis diminished over time. The latter group had a less favorable outcome. The *BLUE* pattern had varying (increasing and decreasing) changes with intermediate outcome. We found a complete fibrinolysis (in vitro clot breakdown) within 20 min, predominantly within a running time of 10 min after starting the in vitro measurement at each testing time point. 4-channel analysis for differences over time during IVT revealed interchangeable ROTEM values for INTEM and EXTEM.

Data on real-time changes in VEM during IVT is scarce. Two main aspects are of interest: first, the identification of potentially predictive markers for clinical outcome and secondly, the time it takes until therapy-induced changes of whole blood coagulation show up in VEM. Both aspects are essential to evaluate VEM as possible point-of-care assessment for IVT.

The largest study (*n* = 171) in our context was published by McDonald et al. [24] They hypothesized that clinical outcome may be predicted by comparing certain TEG baseline parameters to detect a hypercoagulable state. Such patients would potentially respond worse to treatment with alteplase because their clots may have contained higher amounts of fibrin and platelets. Finally, the TEG baseline differences did not correlate with the clinical outcome. However, no dynamic analysis of coagulation parameter changes at multiple time points during IVT was performed.

We hypothesized that certain curve progression patterns, derived from changes of specific ROTEM parameters during IVT, may correlate with early clinical outcome.

In our study, continuous iCL amplitude reduction or even a total clotting suppression (*GREEN* pattern, high responders) was associated with a favorable outcome. This could be explained by an individually sustained effect of alteplase on iCL inhibition in these patients. In contrast, diminishing fibrinolytic response over time (*RED* pattern, low responders) indicated a worse outcome. This in turn could be interpreted as partial recovery of whole blood coagulation during IVT. Patients with varying curve progression patterns (*BLUE* pattern) also benefited from IVT, but to a lesser extent.

The second main question is about timing: coagulation monitoring can hardly be achieved by a continuous measurement throughout 60 min, but by multiple tests during this time interval. Therefore it is important to distinguish between the time an in vitro measurement needs to be completed (=  “running time”) and the testing time point during IVT, when the ROTEM assay is initiated. The running time has to be added to a certain testing time point to determine when the results of the corresponding measurement will become available. For example, when assuming a running time of 10 min, the results of the 15-min measurement will be available 25 min after beginning IVT. With the majority (91%) of iCLs broken down within 10 min, we consider this an appropriate running time for a single ROTEM measurement.

Rowe et al. used TEG to examine a small sample (*n* = 7) before, during and after alteplase administration [18]. The maximum clot lysis and minimal clot strength was already observed in their first measurement after 30 min.

These findings are in accordance with the results of our study, although we used ROTEM. We found a strong fibrinolysis in all iCLs even at the post bolus testing time point, showing up after a running time of 10 min. The early response was relatively uniform in all patients, as nearly all iCLs showed a continuous decrease until the 15-min testing time point. Thereafter, the reaction was more differentiated. That is why we focused on differences between the testing time points at 15, 30 and 60 min.

Nonetheless, the individual extent of amplitude changes during IVT differed considerably among the patients and in the course of time. This explains why absolute cutoff values at a certain testing time point during IVT may not prove useful in general.

Taken the testing time points and running time of our procedure into account, complete results for outcome correlation are available 70 min after initiation of IVT (testing time point at 60 min plus 10 min running time). Independent data from two big stroke registries revealed that the majority (about 87% in both studies) of IVT-eligible patients arrived at the hospital within a time frame of 3 h [25, 26]. Adding 70 min to 3 h would still allow dose adjustments within the standard IVT time window of 4.5 h.

Moreover, risk stratification by means of cerebral imaging recently resulted in extended time frames for IVT up to 9 h [27]. This concept of extending the IVT time frame for selected patients could also apply for in vitro risk stratification with VEM.

Having investigated testing time points and running times, we focused on differences in the 4 channels, recorded in every single measurement. No remarkable differences in iCL characteristics at any testing time point were found in the 4-channel analysis. In particular, INTEM and EXTEM exhibited nearly identical changes of values for the ROTEM parameters MCF, MCF-t and AR10 during IVT.

FIBTEM showed corresponding changes, but differed more from the other test channels. This may be explained by the fact that FIBTEM exclusively represents the fibrin portion of the clot and therefore responds more sensitively to fibrinolytic therapy. Accordingly, iCLs in FIBTEM broke down even earlier than in INTEM and EXTEM, mostly within 5 min of running time. While this fast reaction time in FIBTEM would be an advantage over INTEM and EXTEM, weaker clot firmness in general and a higher probability of a total clotting suppression in vitro are the drawbacks. Thus, FIBTEM can be considered a very sensitive, but likewise diagnostically too inexact assay for point-of-care assessment during fibrinolytic therapy.

As expected for a control channel, APTEM usually showed normalized ROTEM curves after in vitro inhibition of plasmin with aprotinin. Still, in cases of a strong hyperfibrinolysis leading to a total clotting suppression (“zero line” in ROTEM), APTEM also exhibited a prolonged and reduced clot formation. While not adding new insights for our study, this phenomenon could be of interest when observing adverse events like therapy-induced bleedings.

In summary, INTEM, EXTEM and FIBTEM appropriately reacted to fibrinolytic therapy, but both INTEM and EXTEM appear to be most suitable for use in future studies.

Cerebral imaging indicators like infarct location, size of infarction and therapy-induced phenomena like hemorrhagic transformation, brain edema or degree of recanalization provide the most relevant information for mid- and long-term stroke prognosis. ROTEM, as used in our study, may contribute less in this context.

We primarily evaluated ROTEM as a potential point-of-care assessment tool for IVT in the acute stroke management. The fibrinolytic patterns found may allow to distinguish low from high responders in order to perform dosage adjustments of IVT in future trials.

Although not investigated until now, this approach could also be of interest for evaluation of bridging IVT before mechanical thrombectomy.

Our study has limitations. Due to its exploratory design and the limited number of patients included, it has to be regarded a hypothesis-generating pilot study. The findings of this pilot study therefore should facilitate a proper power and sample size calculation for future confirmatory trials.

Our findings are based on in vitro clots, which do not inevitably represent the “true” blood coagulation in vivo. It is not clear if venous blood samples fully depict the arterial system and how changes of whole blood coagulation in vivo are translated to in vitro results. However, given the strong, but differentiated response in ROTEM, it is likely that these in vitro findings also capture the arterial coagulation changes during IVT in vivo and thus allow for clinical outcome correlation.

The baseline NIHSS score was rather low in our sample because patients with higher NIHSS scores predominantly underwent mechanical thrombectomy. As we excluded these patients for methodological reasons, we cannot comment on ROTEM’s ability to detect a successful lysis of a preformed, stroke-causing intra-arterial clot. Achieving this will also depend on local conditions like clot location, length and porosity, which should be addressed in future studies. However, IVT will certainly retain its importance in cases of minor stroke without intra-cerebral artery occlusion. In addition, all patients presented with a clinically relevant disability and response to IVT differed according to distinct ROTEM patterns. In the era of thrombectomy, patients who solely receive IVT will more likely present with lower NIHSS scores, suggesting our sample is more representative.

The results of VEM assays like ROTEM and TEG are not interchangeable [28]. Although total values may differ depending on the applied VEM, both methods are based on the same testing principle and reliably detect fibrinolysis as induced by alteplase [17, 19, 29]. Beside many other advantages, new generation devices like TEG 6S^®^ or ROTEM Sigma^®^ facilitate the clinical use and reduce the need for specially trained personnel such as was required in our study. Hence, further studies should preferably be conducted with these devices.

In conclusion, we found specific dynamic clot patterns during IVT that were associated with different clinical outcomes. The use of ROTEM to monitor the fibrinolytic response of IVT is feasible, reasonably fast and inexpensive. A running time of 10 min and one testing channel (either INTEM or EXTEM) per measurement seem appropriate for point-of-care assessment in IVT. Based on our experience, running ROTEM approximately 15 min after starting IVT at three consecutive testing time points may allow identification of low responders who could benefit from an intensified alteplase treatment in the future.

## Supplementary Information

Below is the link to the electronic supplementary material.

Supplementary file1 (DOCX 243 KB)

## Data Availability

The datasets generated during and/or analyzed during the current study are available from the corresponding author on reasonable request.
