# Supplementary material for: Real-time monitoring of intravenous thrombolysis in acute ischemic stroke using rotational thromboelastometry: a feasibility pilot study
Source: J Neurol. 2022 Jul 19;269(11):6129–38. doi: 10.1007/s00415-022-11271-z (PMC9553850; doi:10.1007/s00415-022-11271-z)
Supplement: Supplementary file 1 — Supplementary file1 (DOCX 243 KB) [file 415_2022_11271_MOESM1_ESM.docx]

**Supplemental Material**

Article title: Real-time monitoring of intravenous thrombolysis in ischemic stroke using Rotational Thrombelastometry: a feasibility study

Journal name: Journal of Neurology

Author names: Alexander Tinchon, Elisabeth Freydl, Robert D. Fitzgerald, Christina Duarte, Michael Weber, Bernadette Calabek-Wohinz, Christoph Waiß, Stefan Oberndorfer

Corresponding author: Alexander Tinchon M.D.

Karl Landsteiner University of Health Sciences, Dr. Karl-Dorrek-Straße 30, 3500 Krems, Austria, Department of Neurology, University Hospital St. Pölten, Dunant-Platz 1, 3100 St. Pölten, Austria

Email: a.tinchon@gmail.com

|  | All patients (n=12) | | | *RED* pattern (n=4) | | | *GREEN* pattern (n=4) | | | *BLUE* pattern (n=4) | | |
| --- | --- | --- | --- | --- | --- | --- | --- | --- | --- | --- | --- | --- |
|  | Min. | Max. | Median | Min. | Max. | Median | Min. | Max. | Median | Min. | Max. | Median |
| **AR10 Difference in %** |  |  |  |  |  |  |  |  |  |  |  |  |
| 15 min - 30 min | -100,0 | 62,3 | -31,9 | 7,2 | 28,6 | 8,2 | -100,0 | -31,9 | -100,0 | -45,5 | 62,3 | -25,0 |
| 30 min - 60 min | -72,3 | 870,3 | 67,4 | 55,6 | 870,3 | 122,7 | -31,6 | -31,6 | n.c. | -72,3 | 77,6 | 70,8 |
| **MCF Difference in %** |  |  |  |  |  |  |  |  |  |  |  |  |
| 15 min - 30 min | -100,0 | 70,0 | -6,3 | 5,0 | 70,0 | 5,6 | -100,0 | -12,5 | -100,0 | -44,4 | 15,8 | -15,4 |
| 30 min - 60 min | -72,7 | 119,0 | 27,8 | 29,4 | 119,0 | 48,9 | -14,3 | -14,3 | n.c. | -72,7 | 27,8 | 18,3 |
| **MCF-t Difference in %** |  |  |  |  |  |  |  |  |  |  |  |  |
| 15 min - 30 min | -100,0 | 25,8 | -6,7 | 6,1 | 13,7 | 7,2 | -100,0 | -6,7 | -100,0 | -23,5 | 25,8 | -6,6 |
| 30 min - 60 min | -24,0 | 464,0 | 35,5 | 0,0 | 464,0 | 91,4 | -24,0 | -24,0 | n.c. | -0,9 | 43,2 | 30,6 |

**Table S1**

Percentage changes of the ROTEM parameters AR10, MCF and MCF-t in the 15-30 minutes and the 30-60 minutes measurement, sorted by patterns. N.c.=not calculable (n=1).


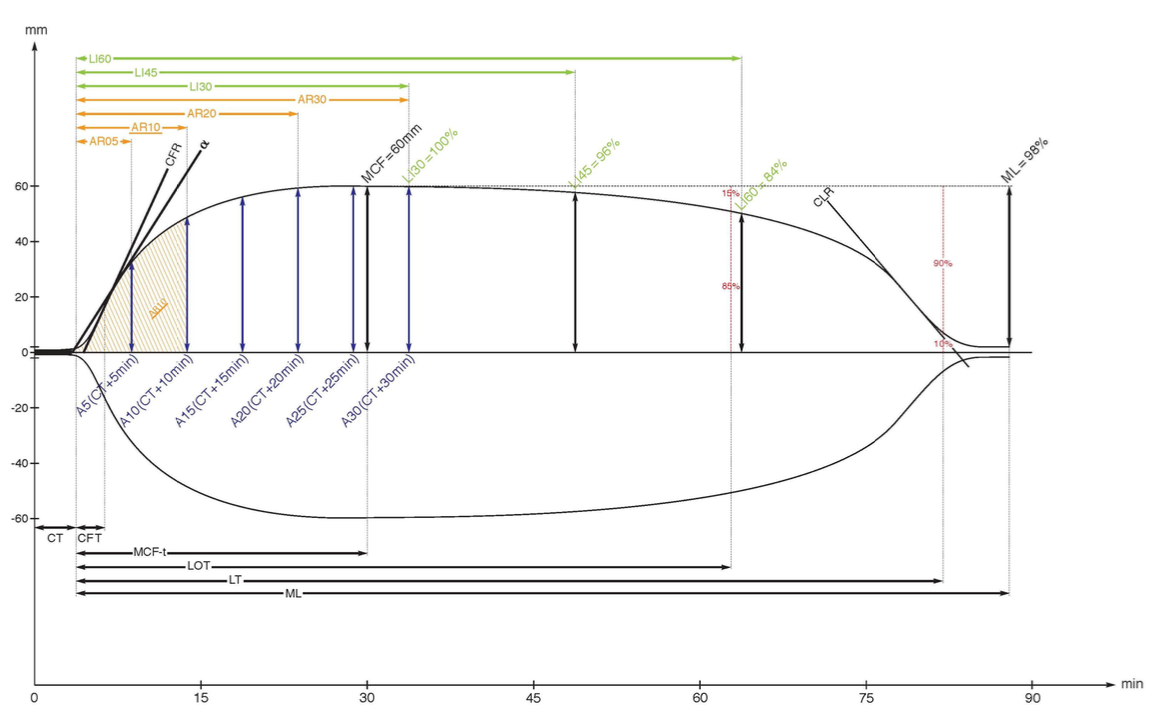


**Figure S1**

Overview of standard ROTEM parameters. CT=clotting time; CFT=clot formation time; Ax=amplitude after x minutes; ARx=area under the curve after x minutes; MCF=maximum clot firmness, MCF-t=time to maximum clot firmness.
